# Second-Level Nurses' Experiences of Workplace Violence: A Scoping Review

**DOI:** 10.1155/2023/6672952

**Published:** 2023-11-17

**Authors:** Gwenn Recla-Vamenta, Lisa McKenna, Ewan McDonald

**Affiliations:** ^1^School of Nursing and Midwifery, La Trobe University, Bundoora, Victoria 3086, Australia; ^2^Nursing and Midwifery, College of Sport, Health and Engineering, Victoria 8001, Australia; ^3^Bendigo Health, Bendigo, Victoria 3550, Australia

## Abstract

**Aim:**

To synthesise and map what is known about second-level nurses' workplace violence experience.

**Background:**

Workplace violence has become a topic of focus in nursing over recent years. Research demonstrates that there is a growing body of literature focusing on first-level nurses' workplace violence experiences, but those of second-level nurses, a crucial component of the health workforce in many countries, have not been well explored. *Evaluation*. A scoping review was conducted from January 2000 up to March 2022 using Arksey and O'Malley's framework and databases, including ABI/Inform Collection, CINAHL, DOAJ, EBSCOhost, and ProQuest Central. *Key Issues*. Eighteen studies were included in the final review. The review identified three key findings related to second-level nurses' workplace violence experience. (1) They were found to have experienced physical and non-physical violence. Among non-physical violence, they reported experiencing bullying, mobbing, sexual harassment, racial discrimination, nurse-to-nurse conflict, and electronic abuse. (2) They were more likely than first-level nurses to experience physical violence, and (3) often data from second-level nurses were combined with those of first-level nurses; hence, it was difficult to identify the specific experiences of second-level nurses.

**Conclusion:**

The review contributes to new knowledge highlighting the second-level nurses' workplace violence experiences worldwide. The review indicated that there are gaps identified and there is a need for greater understandings of workplace violence in second-level nurses to understand the scope of their problem and the nature of their experiences. *Implications for Nursing Management*. Nurse managers play a critical role to develop and implement effective policies and evidence-based interventions to improve the working conditions of the second-level nurses. The results of this current review can be used to guide nurse managers and organisations in providing adequate support to reduce and prevent WPV and advocate for a positive workplace culture.

## 1. Background

Workplace violence (WPV) and harassment are defined as “a range of unacceptable behaviours and practices or threats thereof, whether a single occurrence or repeated, that aim at, result in, or are likely to result in physical, sexual, or economic harm and include gender-based violence and harassment” [1]. WPV is not a new phenomenon. Accordingly, violence against the workforce is a serious problem in healthcare organisations, reportedly affecting up to 95% of healthcare professionals internationally [2].

WPV is a multifactorial and complex problem. It poses a significant threat to the health, safety, and wellbeing of healthcare professionals [3], having been linked with depression [4], anxiety symptoms [5], sleep problems [6], burnout, and mental fatigue [7], which is indicated to have negative consequences for the healthcare workers' productivity [8], retention [9], and the quality of care they provide to their patients [10]. In 2014, estimated costs of WPV perpetrated by hospital patients or patient visitors in the US were $94,156 annually ($78,924 for treatment and $15,232 for indemnity) for a total of 106 (2.1%) of 5,016 hospital system nurses who reported WPV injuries [11]. The estimate does not capture the hidden costs of WPV-related incidents, including emotional pain, depression, isolation, and anxiety [12].

WPV in the healthcare industry is recognised as a significant workplace health and safety concern, with the nursing workforce identified as the profession at most significant risk of being assaulted [13]. It remains a persistent source of distress among nurses, irrespective of the contexts and settings in which they work [14]. Furthermore, WPV is not limited to hospital settings but extends to all healthcare work environments and geographical locations, including rural, remote, metropolitan, international, community, mental health, and aged care, where nurses have more significant contact with patients and members of the community [15, 16].

It is asserted that WPV against healthcare professionals remains an underreported and pervasive problem that has been tolerated and largely ignored [3]. Subsequently, it also highlights challenges for researchers and highlights the pressing need for further research evidence to uncover universally applicable risk reduction methods [3]. The nursing profession is experiencing substantial nursing shortages due to economic pressures and the impact of an ageing population across the world, a crisis recognised before the COVID-19 pandemic [17]. Consequently, this has prompted to a progression of healthcare workforce skill mix and adaptation to new roles to respond to changing needs [18]. In many countries, there are two levels of nurse: the Registered Nurse (RN), or First-Level Nurse (FLN) and Second-Level Nurse (SLN). In Australia, RNs are educated in a three-year Bachelor of Nursing program, while SLNs are educated in the Vocational Education and Training (VET) sector in a two-year Diploma of Nursing pathway [19], and they have different education levels elsewhere. Across countries where these roles exist, second-level nursing roles and titles vary widely, including Nursing Associate (NA) in the United Kingdom (UK), Licensed Practical Nurse (LPN) and Licensed Vocational Nurse (LVN) in the USA, Registered Practical Nurse (RPN) and Licensed Practical Nurse (LPN) in Canada, and Enrolled Nurse (EN) in Australia, New Zealand (NZ) [20], Singapore [21], and Hong Kong [22]. SLNs' roles and scope of practice differ among countries. In most cases, they work in various healthcare settings under the supervision of RNs, conducting patient observation, providing basic care, attending to patients' activities of daily living, such as hygiene care, and assisting support during rehabilitation [23].

A hierarchical healthcare workforce structure exists between first level and second-level nurses, which potentially marginalises SLNs. They are situated low in the healthcare hierarchy while also at the coalface of patient interaction. RNs are perceived to provide critical thinking compared to SLNs' task-orientated, hands-on patient care [24]. Furthermore, RNs are given higher ranking status, and contrastingly, SLNs are perceived as subordinated taskdoers, underpaid, undervalued, and low status [23]. Roberts [25] identified that nurses exhibit oppressed group behaviours (OGB). The word “oppression” in nursing is characterised as powerlessness and submissiveness [26]. The model of OGB theory in nursing may help explain oppressed nurses' display of submissive behaviours in response to the domineering and powerful groups of physicians and hospital administrations [27]. Roberts [25] explained that oppressed groups of nurses manifest collective lack of self-esteem. Furthermore, they develop submissive-aggressive behaviour syndrome. Subsequently, when nurses are not able to control self-hatred, low self-esteem, and dislike for other nursing staff, eventually these kinds of behaviours present negative consequences including horizontal violence and lateral violence [25].

Given the difficult circumstances around SLNs in the nursing workforce and limited research on SLNs in Australia [28–30], the UK [23], Canada [31], and Singapore [32], we sought to understand their experiences of WPV. To date, there has been a growing body of literature focusing on RNs' WPV experiences, yet that of SLNs has not been well explored. Hence, this scoping review sought to map and synthesise available international evidence on SLNs' unique experiences of WPV.

### 1.1. Scoping Review Objective

To synthesise and map available international evidence and what is currently known about SLNs' specific WPV experiences.

## 2. Methods

A scoping review is defined as a “form of knowledge synthesis that addresses exploratory research aimed at mapping key concepts, types of evidence, and gaps in research related to a defined area or field by systematically searching, selecting, and synthesising existing knowledge” [33]. A scoping review is useful for examining emerging evidence, identifying knowledge gaps, and providing a rigorous and transparent method for identifying and mapping available evidence [34]. The current scoping review was conducted based on the methodological framework by Arksey and O'Malley [34] and included the five stages of identifying the research question: identifying the research question, identifying the relevant studies, study selection, charting the data, and collating, summarising, and reporting the results [34]. Levac et al. [35] further clarified and enhanced Arksey and O'Malley's [34] framework and proposed recommendations for each stage of the scoping study framework, emphasising considerations for advancement, application, and relevance in maximising the usefulness and rigour of scoping study findings within healthcare research and practice.

### 2.1. Research Question

The research question was: “What are SLNs' experiences of WPV?”

### 2.2. Relevant Studies

A preliminary search of the literature was undertaken to identify relevant studies in investigating SLNs and WPV internationally (Table 1). To achieve rigour, several consultations from the research librarian were undertaken. Using the search terms, “Second Level Nurs^*∗*^,” “Enrolled Nurs^*∗*^,” “Endorsed Enrolled Nurs^*∗*^,” “Licensed Practical Nurs^*∗*^,” “Licensed Vocational Nurs^*∗*^”; (b) workplace violen^*∗*^, “horizontal violen^*∗*^,” “lateral violen^*∗*^,” “vertical violen^*∗*^,” “upwards violen,” “nurse-to-nurse” conflict', “mobbing,” “incivility” and “nurses eating their young,” six electronic databases were searched: ABI/INFORM Collection, Cumulative Index to Nursing and Allied Health Literature (CINAHL), Directory of Open Access Journals (DOAJ), EBSCOhost Human Resources Abstracts, Medline ProQuest, and ProQuest Central. Keywords and subject headings were modified to suit the requirements of the databases. Keywords using the truncation symbol (^*∗*^) to broaden search terms within title and abstract fields were used in an initial search. In addition, Boolean operators or connecting words “OR” and “AND” were used to limit, broaden, or define searches within and between categories.

### 2.3. Study Selection

Following the Preferred Reporting Items for Systematic Reviews and Meta-Analyses (PRISMA) guidelines [36], inclusion criteria included primary research comprising qualitative, quantitative, and mixed methods studies, with full-text availability. Studies published between 1 January 2000 and 3 March 2022 in English language were included. This time frame was chosen based on when the need to advance EN education in Australia was recognised [37] up until the search date. Furthermore, in the UK, it was in 2002 when the Nursing and Midwifery Council (NMC) was established to ensure safe practices and professional standards were achieved [38]. In addition, studies included participants working as SLNs who had experienced WPV within public hospitals, private hospitals, aged care, primary/community, and other healthcare settings. The exclusion criteria included reviews of research, discussion papers, editorials, conference abstracts, and book reviews. Furthermore, studies with no data on SLNs, no available full text, or incorrect study design were excluded from the review.

A total of 1145 studies were identified from an initial database search, and titles and abstracts were imported into EndNote then uploaded to Covidence [39] for screening. In addition, eight studies were added after hand-search in Google Scholar. A total of 134 duplicates were removed. In total, 1011 titles and abstracts were initially screened independently by three authors based on inclusion and exclusion criteria.

**Figure 1:**
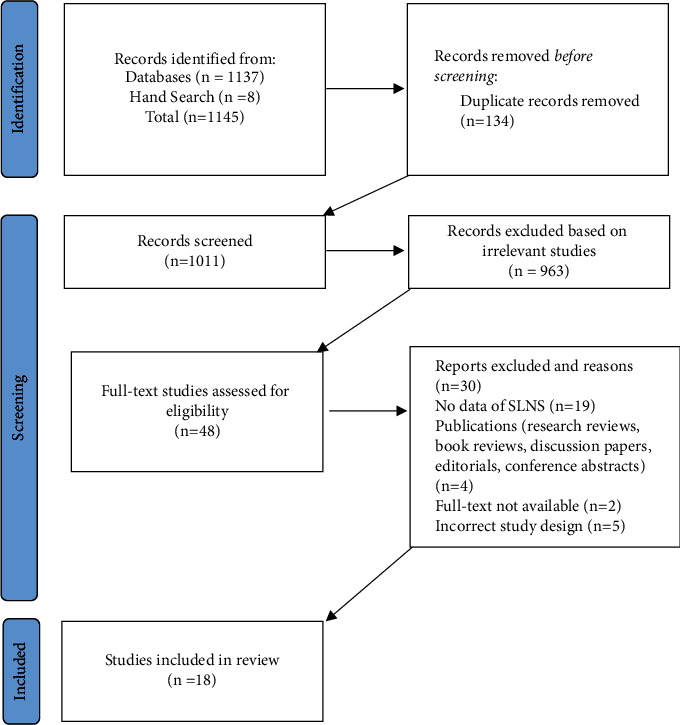
Study flow diagram using Preferred Reporting Items for Systematic Reviews and Meta-Analyses (PRISMA) flowchart [36].

A total of 963 studies were excluded at this stage. Of the remaining 48 studies subjected to full-text review, 30 were excluded as no specific data could be extracted on SLNs, they were wrong publication type, full text was not available, or the study design was incorrect. After rigorous examination, 18 studies were included in the final critical appraisal and data extraction review. A small number of conflicts arising were resolved during team discussion (Figure 1).

### 2.4. Data Charting

Quality appraisal was undertaken using the Joanna Briggs Institute (JBI) [40] critical appraisal tools. While not always included in scoping reviews, critical appraisal was conducted on all studies to assess overall quality [41]. Criteria in judging the quality of the studies are described by evidence that is trustworthy, applicable to practical settings, consistent, and unbiased, irrespective of whether a qualitative or a quantitative method is employed [42]. While some studies had low scores, the authors' intention was not to remove studies but to assess the overall quality of the existing knowledge base. Hence, no studies were removed based on quality appraisal. The following data were extracted from the included studies based on JBI guidelines for scoping reviews [43]. Data were extracted from the studies: author, year, country, aims, study design, data collection, data analysis, participants, research location, key findings, results, and study limitations.

### 2.5. Collating, Summarising, and Reporting the Results

The primary author extracted data and undertook the collation process of all included studies, and to ensure accuracy, the two other authors confirmed data extraction. Any inconsistencies were resolved during team discussion. The results were synthesised by summarising study details and findings. Tabulation of extracted data of study characteristics was utilised, and key study findings were compared [44].

## 3. Results

### 3.1. Description of the Studies

Table 2presents the characteristics of final included studies. Of the 18 studies, the majority were conducted in North America (*n* = 12, 66%), followed by Europe (*n* = 3, 16.66%), Australia (*n* = 2, 11.11%), and South Africa (*n* = 1, 5.55%). There were 17 published papers and one doctoral dissertation. Across the studies, authors employed a diverse range of research study designs. Eight adopted cross-sectional designs [47, 50, 53, 56, 58–61]; three had qualitative designs [48, 49, 55]; two were case-control studies [54, 57], and there was one each of quantitative [45], phenomenological [46], quasi-experimental [51], cohort [52], and retrospective prevalence studies [62]. Workplace locations of reported WPV included emergency, medical-surgical, acute care, mental health [46, 48, 54–59], nursing homes, and residential care facilities [45–48, 54, 56–59, 61]. Furthermore, two studies explored education preparation training to combat WPV [51, 58].

Numbers of participants identified as SLNs in studies ranged significantly from two to 4076. Regarding research location, three studies were undertaken across multiple healthcare facilities and clinical areas; four in nursing homes and residential care facilities; multiple studies were drawn from state or national databases; and one from a college department. Four included studies were published within the last five years (2018 to 2022), while the remaining 14 were published before 2017. Three of the 18 studies focused explicitly on SLNs' experiences [50, 52, 53], while in the remaining 15, participants were a mix of RNs and SLNs [45–47, 49, 51, 54–62] or part of a diverse range of healthcare disciplines [48].

Five studies analysed written narratives of SLNs' lived experiences [46, 48, 49, 55, 56]. Although the studies integrating qualitative data were pivotal to significant contributions by providing rich, thick, and nuanced data, issues were encountered, including dependability and confirmability. There were also challenges in unpacking and extracting specific data for SLNs. For example, in the two Canadian studies, one focused on participants' ethnicities and racial backgrounds but did not explicitly specify the number of SLNs [46], while the other did not mention the risks of WPV in a specific healthcare profession [48]. Likewise, in research adopting quantitative methods, self-administered surveys, issues of reliability and potential biases, and descriptions of participants' work locations were sometimes not disclosed.

### 3.2. Specific Aspects of SLNs' WPV Experiences

#### 3.2.1. Nature of Violence/Conflict

*(1) Physical Violence*. Physical violence is when an individual is hit, slapped, pushed, choked, grabbed, sexually assaulted, and exposed to physical contact to injure or harm [54]. Physical violence represented one of the most common types of WPV for SLNs reported, ranging from 10.6% to 76% [45–48, 54–58, 60, 62] (Table 3). Two studies mentioned the types of physical violence injuries frequently reported as bruises, contusions, temporary discolouration, lacerations, and punctures [54, 57]. The remaining studies reported physical violence according to broad definitions of physical violence, without specific details. Two studies confirmed that SLNs working in nursing homes or residential settings reportedly experienced physical violence greater than those working in other healthcare settings [45, 47]. For example, SLNs and other nursing staff (RNs, Nurses' Aides) working in Swedish nursing homes revealed significantly higher reported WPV incidents (*P* ≤ 0.01) compared to those working in other healthcare settings [45]. Similarly, in Canada, SLNs and Health Care Assistants, working in dementia units reportedly experienced considerable aggressive acts compared with those working in Alzheimer's units (mean = 2.4 vs. mean = 0.9, *p* < 0.001) [47]. Most physical violence incidents reported clients/patients [54, 57, 60] or co-workers [60] as perpetrators.

*(2) Non-Physical Violence*. Non-physical violence can be classified as verbal abuse, threats, ironic language, derogatory glances, and provocative or aggressive body language [63, 64]. Non-physical violence experienced by SLNs was examined in numerous studies, with reported prevalence rates ranging from 12% to 87.9% [45, 46, 48, 50, 51, 53–58, 60] (Table 3). The studies reported non-physical violence according to the broad definition of non-physical violence. Compared to other categories of nurses and healthcare workers involved, two studies revealed that non-physical violence was more likely reported by SLNs [54, 57]. Perpetrators associated with non-physical violence were patients/clients [54, 57], physicians, and other employees [57]. Psychological abuse [45, 48, 55], verbal abuse or aggression [46, 47, 55, 58, 60], emotional abuse [47], bullying [50, 51, 53], and mobbing [61] were also identified.

Three studies discussed SLNs' bullying experiences in the workplace [50, 51, 53], with varying experiences. Participants self-identified as being bullied, and the frequency of bullying behaviours reportedly ranged from at least one to 20 bullying behaviours and reported bullying acts ranged from never, now and then, monthly, weekly to daily [51] The moral component of authentic leadership (AL) was a major determinant of overall workplace bullying (*β* = −0.59, *p* < 0.001), person-related bullying (*β* = −0.70, *P* < 0.001), and physical intimidation (*β* = −0.58, *p* < 0.001) [53]. Unmanageable workloads, being ordered to work below the level of confidence, withholding information, having opinions and views ignored, and having responsibilities removed were cited as the most frequently reported negative acts of bullying [51, 53]. In contrast, the most reported perpetrators were peers, followed by supervisors [53].

A study from Swiss nursing homes was conducted examining the prevalence of workplace mobbing incidents, which was reported to be higher among SLNs and RNs compared to the unregulated healthcare workers (Nurse Aides) and other professionals (5%, 5.9%, and 2.6%, respectively) [61]. Mobbing is defined as repeated, unwarranted behaviour aimed at an employee or group of employees evoking a threat to health and safety [65], occurring at least every week over six months or more [66]. It is deliberate, systematic, and continuous harassment, similar to bullying, which involves physical violence or verbal harassment aimed at disturbing harm to the person being targeted [67]. Mobbing is directly correlated to job satisfaction and intention to leave (*p* < 0.001) among hospital nurses (RNs, SLNS, Assistant Nurses, and Nurse Aides) [61]. Overall, the prevalence of mobbing experiences for Swiss healthcare workers was relatively low [61].

Three studies examined sexual harassment, two from Canada [46, 47] and another from the USA [57], with SLNs reportedly experiencing this ranging from 7% to 22%. One study comprising a mixed nurses' population (FLNs and SLNs) did not explicitly specify the exact number of SLN participants; however, findings revealed that five ethnic minority nurses reported sexual harassment compared to one white Canadian nurse [46]. Another study indicated that SLNs experienced higher incidence of forced sexual harassment compared to Health Care Assistants (22.2%, 5.5%, *p* = 0.046) [47].

Two Canadian studies examined racial discrimination by exploring FLNs and SLNs' experiences of work conflict with patients and their family members, primarily focusing on ethnic minority nurses compared to white Canadian nurses [46], and another exploring cultural and racial differences [48]. The visibly ethnic minority nurses, especially blacks, reported significant discrimination due to the colour of their skin, language barriers, accent, or perceived lack of competence [46]. Two of seven qualitative narrative comments were discussed in that study, focusing on SLNs involving derogatory and disrespectful comments towards them.

One Australian study explored nurse-to-nurse or intra-professional conflict, and reportedly, many SLNs experienced bullying, stress, and harassment from RNs in the workplace [49], with common themes emerging as the scope of practice, teamwork, and team conflict. SLNs reported that the terms “workloads” and “teamwork” were used interchangeably as perceived realities of unfair work distribution between SLNs and RNs, the “us and them” phenomenon. In the theme of the scope of practice, participants were critical of wage gaps between themselves and RNs. The added responsibility of medication endorsement was initially perceived as a positive milestone for Endorsed SLNs, which would alleviate some traditional responsibilities and the SLNs' given extended responsibilities and scope of practice. The SLN role traditionally focused on direct patient care activities and routine nursing tasks such as attending hygiene care and simple wound dressings [49]. Furthermore, the study explored the sense of powerlessness of SLNs in maximising their skill sets around medication administration. SLNs identified an inability to practise their skills depending on the ward and which RNs they worked with. The researchers concluded that SLNs' scope of practice was heavily influenced by whom they built alliances or partnerships, years of working experience, and sometimes understaffing issues [49].

Electronic (e-mail) abuse was also explored in one study, with 7.1% of SLNs reported being the least victims of e-mail abuse compared to other nursing professions [60]. Electronic abuse is defined as “a statement of behaviour that is reasonable for a worker to interpret as a threat or abuse via e-mail” [60]. Examples include bullying, defaming, harassing, interrogating, accusing, and blaming [60]. Significant relationships were found between electronic abuse and education (0.001) and electronic abuse and years of experience (<0.001) [60]. In this study, nurses with diplomas were reported to be the least electronically abused compared to other nursing professionals who had achieved academic attainments (bachelor's or master's degrees and “other”) [60].

### 3.3. Long-Term Impacts and Outcomes

Long-term impacts and outcomes of WPV for SLNs, patients, and organisations can be classified into attitudinal, behavioural, health, financial, and patient care impacts.

Two studies explored attitudinal impacts, including low job satisfaction and intention to leave [54, 61] and “normalised” or “part of the job in nursing” [48]. Five examined behavioural including powerlessness, sadness, defeat, guilt, shame and low-self-esteem, anger, fear, anxiety, frustration [45, 48, 57], absenteeism, and presenteeism [54, 61] (Table 3).

Multiple studies discussed the health impacts of SLNs from WPV; these include emotional and physical impacts. Emotional impacts resulting from WPV incidents include post-traumatic stress disorder (PTSD), which is described as a severe and common result of violence, and common symptoms include anxiety and difficulty sleeping [48]. In addition, stress and suicidal ideation [48, 50], fatigue, difficulty concentrating, tiredness, lack of energy [57, 61], depression [57], flashbacks, nightmares, and hallucinations [54, 57, 61] were explored in the studies. Physical impacts include physical injury, disability, reduced quality of life [54], generalised body pains [54, 61, 62], and headache/head pressure [57, 61].

Three studies also explored the financial impacts of WPV, including reduced productivity and lost employee work hours [54, 62], increased turnover, decreased retention, and nursing shortage [54, 56], salary replacement dollars for overtime and agency staff [62], counselling cost [54], potential organisational ramifications from lawsuits, and negative publicity with potential negative impact on staff recruitment and retention [62].

Patient care impacts included that delivery of patient care could lead to increased documentation and medication errors, staff focus being shifted from work activities to discussion of incidents, and consequently, decreased attention to patient care needs [56, 62] and failure to implement protection through adequate staffing, programs, engineering controls, and environmental design [48].

### 3.4. Actions and Coping Strategies

A range of actions and coping strategies were identified in the included studies, which participants offered based on their own experiences (Table 3). They can be categorised according to primary, secondary, and tertiary prevention strategies [48].

Primary prevention strategies are aimed at preventing WPV from occurring. Multiple studies explored primary prevention strategies [46–49, 53, 55, 58, 59]. Such strategies include enhanced hospital security [46, 48], “switching” with other nurses who had better work relationships with patients or working in pairs for patients who had been flagged as uncivil [46], seeking advice from colleagues, family members or friends, or talking to patients' family members [46, 49, 55], adopting zero-tolerance policies, increasing staffing, and using personal alarms [48]. Other primary prevention strategies included WPV training programs [48, 58] and supportive organisational environments [53].

Secondary prevention or early prevention strategies are actions to prevent violence at early signs of violence. Several studies investigated secondary prevention, including encouraging and simplifying incident reporting processes and using the criminal justice system [48] and repressive or sanctioning interventions [47].

Tertiary prevention strategies are actions taken when violence is occurring or after it has occurred to prevent or reduce the potential for physical and psychological harm to parties involved and to inform subsequent primary and secondary prevention strategies. Tertiary prevention strategies were mentioned in multiple studies and included teamwork, open communication [53], and policy-based strategies and support [46, 47]. One study suggested adopting a “radical” mechanism of leaving the ward, manipulating nursing staff rosters, or “shutting down” to avoid further conflicts [49].

## 4. Discussion

The scoping review sought to explore WPV among SLNs. This was found to be present worldwide, and settings included hospitals, communities, nursing homes and residential settings, metropolitan and non-metropolitan areas, and diverse nursing specialty areas. To the authors' knowledge, the current review is the first comprehensive review that has attempted to explore WPV specific to this group. Eighteen studies were included, and more than half were conducted in North America, indicating that this has been a research focus in that country but not necessarily in others globally. Overall, included studies were dominated by research in high-income countries, including North America, the USA (38.8%) and Canada (27.7%). Conversely, there was much less in European countries, including Sweden, Norway, and Switzerland (5.55% respectively), followed by Australia (11.11%) and Africa (5.55%). There were no included studies from Asian, African, or South American countries. Hence, further research is needed from such countries.

The scoping review highlights scant international evidence around SLNs' specific WPV experiences. Across the studies reviewed, evidence of reported prevalence rates is unclear. Reporting prevalence rates experienced by SLNs revealed significant variations of WPV, physical violence (10.6% to 76%) and non-physical violence (12% to 87.9%). Due to differences in study methods and populations studied, it is difficult to compare studies.

One possible explanation for the broad range of prevalence rates is the lack of consensus on definitions of WPV. Despite efforts to document international data on WPV, studies have indicated a lack of consistency in defining WPV across countries, and appeals have been made to standardise definitions [3, 68, 69]. In the current review, most included studies used a broad definition of violence. Considering that experiences of WPV are primarily distinctive, it is possible that researchers' goals in conducting the studies is to capture the overall picture of all violent incidents, relying on an individual's interpretation of violence, thus using a broad definition of violence [45]. Furthermore, in the current review, the difference behind a wide range of prevalence rates was the definition of bullying/mobbing: “never, now and then, monthly, weekly, or daily” [51] or “occurring at least every week, over six months, or more” [66]. Therefore, more research is needed to understand better explanations of prevalence estimates and how to support this group of nursing professionals effectively.

Regardless of the limited comparability of research studies that have examined SLNs' WPV experiences, some findings share similarities with prevalence rates with other categories of nurses. For example, there is a reported wide variation in prevalence rates of horizontal violence reportedly experienced by RN graduates (working in their first year of practice) [70]. Horizontal violence, also known as lateral violence, bullying, or incivility, comprises a group of negative and harmful behaviours among peers [71]. RN graduates reportedly experienced a wide variation of horizontal violence at different times working in their first year of practice in countries such as Iran (*n* = 278, 46%) [72], Canada (*n* = 242, 98%) [73], China (*n* = 357, 77%) [74], and South Korea (*n* = 312, 47%) [75].

Similarly, our findings are comparable with nursing students' varying prevalence rates. In Italy, nursing students reportedly experienced physical or non-physical violence (*n* = 346, 34%) during clinical activities [76]. In Pakistan, nursing students (*n* = 309, 73.1%) reportedly experienced WPV in the last 12 months [77]. In a modified self-report online survey in the USA, nursing students (*n* = 126, 100%) reportedly experienced WPV in their clinical placements [78]. Furthermore, more than half of nursing students in Australia (*n* = 55, 58% of second year; *n* = 32, 57% of third-year students) [79] and China (*n* = 486, 52%) [80] reportedly experienced physical or non-physical violence.

The findings suggest that SLNs share commonalities with graduate RNs and nursing students experiencing this phenomenon. It is possible that SLNs, like graduate RNs and nursing students, are vulnerable and are at higher risk to WPV. Jafree [77] stipulates that nurses have greater chances of experiencing WPV and this may be due to low-ranked status, working long hours in the hospital, exposure to patients, less experience, and less likely to report WPV. Previous studies confirm that graduate RNs [73, 75] and nursing students [76–79] are particularly vulnerable and prone to WPV. Graduate RNs are considered lower-ranked nursing category and prone to WPV exposure due to their relatively limited work experience, less confidence in their new role, and lack of awareness of the implicit rules of the work environment [69]. Researchers have also suggested from a retrospective survey on an Italian experience that nursing students were exposed to WPV during their clinical placements due to their lack of experience, changes of wards, and new environment and patient interaction; thus, they were vulnerable to patient-to-nurse violence [76]. Based on this current review, there is a need for more research exploring SLNs' specific issues and reporting of prevalence experienced by SLNs which is currently insufficient on which to draw conclusions.

The results of this current review presented limited published studies of SLNs and WPV, revealing small sample sizes of SLNs and unclear numbers of SLNs in many of the studies. The key findings from SLNs were often found to be mixed with those of FLNs; therefore, it was challenging to capture their specific WPV experiences. Undoubtedly, this review underscores the existence of WPV. It is possible that SLNs experienced more physical violence and non-physical violence in their workplace. Healthcare professionals, specifically nurses, are among the most exposed to WPV [81]. The nursing workforce comprises frontline workers, and patients spend more time with nurses than other healthcare professionals, thus increasing the potential for violence [82]. Findings from a previous quantitative review revealed that most physical and non-physical violence is committed by patients and their families/friends, and non-physical violence is often committed by staff members such as other nurses [83]. Based on this current scoping review, despite significant research on WPV in nursing, the research mainly focuses on FLNs or mixed nursing populations. SLNs are an essential nursing workforce in the healthcare industry; therefore, it is crucial to explore more about their unique WPV experiences.

Compared to other categories of nurses and healthcare workers included in studies, five reported that SLNs were most likely to be physically assaulted [48, 49, 52, 55, 58]. One study in the current review indicated that SLNs were more likely than RNs to experience physical violence [57]. Incidents of physical violence against nurses are widespread in hospital environments [83] and reportedly exacerbated in aged care facilities [84]. Consistent with other research findings, nurses were subjected to significant WPV and aggression. According to the World Health Organisation (WHO) [85], up to 38% of nurses worldwide experienced physical violence throughout their nursing careers. In a systematic review and meta-analysis conducted in the Southeast Asian and Western Pacific Regions, nurses working in private and public hospital environments, specifically those working in psychiatric and emergency departments, are reportedly exposed to higher levels of WPV [86]. In a descriptive, quantitative study conducted in a teaching public hospital in Brazil, emergency nurses reportedly experienced verbal abuse (*n* = 21, 38%), mobbing (*n* = 14, 25.4%), and physical violence (*n* = 6, 11%) [87]. A meta-analysis study conducted to assess the incidence of violence against Chinese nurses indicated that the 12-month incidence of WPV was 71% (95% CI: 67%–75%), and the incidence of verbal violence (63%) (95% CI: 58%–67%) was the most common type of violence [88].

Based on the findings of the current review, there are potential reasons why SLNs might encounter higher rates of physical violence than RNs. Butcher and MacKinnon [20] contend that contextual factors, such as the ambiguous scope of practice for SLNs and RNs in the workplace and the nursing profession's hierarchical relationships, played a critical role in fostering tension and SLN-to-RN conflict. International studies have indicated an ambiguous scope of practice between SLN and RN roles [23, 89–93]. Furthermore, SLNs are perceived to be of lesser status in the nursing hierarchy [23, 89–91, 93], which tend to separate SLNs and RNs. Additionally, SLNs are perceived as undervalued, unless they transitioned to becoming RNs [30]. Consequently, the lack of clear distinction of roles between SLNs and RNs, expanded scopes of practice, role confusion, perceived inequity in workload distribution, and lack of respect could potentially contribute to SLNs being exposed to intra-professional conflict [49]. Based on this discussion, there is insufficient research exploring SLNs' typically unique WPV experiences. Hence, further research is needed to understand the nuances of SLNs' specific workplace issues and the short- and long-term effects of exposure to WPV, particularly in the healthcare settings where it is an important concern.

The current review indicated that SLNs reportedly were more prone to experiencing WPV in various workplace settings, including emergency, medical, surgical, acute care, mental health, nursing homes, and residential care facilities. The findings are comparable to other studies highlighting locations where WPV towards nurses occurs. For example, in a cross-sectional study conducted in Italy, emergency nurses reportedly experienced widespread verbal abuse and physical assault [94]. In a cross-sectional study conducted in psychiatric hospitals in China, nurses reportedly experienced a high prevalence of verbal and physical violence [95]. In Australia, according to the New South Wales Nurses and Midwives Association Survey, it was found that more than 90% of 282 nurses working in aged care facilities had experienced at least one incident of WPV (physical and non-physical) during the previous 12 months from residents, relatives, and visitors [96]. In a systematic review and meta-analysis study, the global prevalence and predictors of WPV committed by patients or visitors towards healthcare workers are high, with disparities in geographical locations, workplace settings, work schedule, and occupations [64]. This comprehensive study's key findings reported that the prevalence of any type of WPV against healthcare workers is high, specifically in psychiatric and emergency settings in Asia, North America, and Australasian countries. Among occupations, nurses (*n* = 138,857, 44.9%) reportedly experienced the highest of any type of WPV, physical and non-physical violence, compared to doctors (*n* = 44,537, 40.1%) and other healthcare professionals (*n* = 18,824, 41%) [64]. Based on these findings, initiatives and effective approaches are needed to mitigate WPV incidents.

The current review indicated that SLNs, like FLNs of ethnic minority backgrounds, reportedly experienced racial discrimination. Despite relatively small data and the challenges of extracting data for SLNs, this review flags that visible minorities experienced racial inequality where patient and patient family members reportedly prefer white nurses. This is indicative of a racial hierarchy where white nurses are perceived as competent and superior, and ethnic minority nurses as inferior [46]. Similar findings indicated that the experiences of racial discrimination were common among minority and ethnic nurses compared to white nurses. In a cross-sectional study conducted from a survey of 528 UK nurses, black, Asian, and minority ethnic (BAME) nurses reportedly experienced three times more discrimination than white nurses and midwives [97]. The study's key finding indicated that bullying and discrimination were substantially associated with higher burnout and higher burnout was linked with poorer perceptions of patient safety on the individual and ward level [97]. An anonymous online questionnaire survey conducted to explore the experiences of NZ RNs of Chinese ethnicity during the COVID-19 pandemic indicated that 47.06% of the 51 nurses reportedly experienced racial discrimination, workplace bullying, and judgement [98]. A study from the National Commission to Address Racism in Nursing [99] demonstrated that racism in nursing was problematic, with approximately 56% of 5,623 nurses reporting that racism affected their wellbeing. Thurman et al. [100] stipulate that contributing factors linked with nurses' deteriorating mental wellbeing include chronic staff shortages, increasing workload, poor renumeration, bullying, harassment, discrimination, and racism in the workplace. Based on the current review findings, it is therefore crucial that nurse managers and organisations must invest in the SLNs' overall wellbeing by promoting an inclusive nursing culture and culture of change. Creating a culture that values inclusion requires a critical review of the culture of an organisation. Nurse managers need to practise effective leadership by ensuring inclusive cultures within organisations, manifested by no tolerance for negative or toxic cultural norms, such as bullying and WPV, which can profoundly impact workplace behaviour [101]. An inclusive culture is also achieved when cultural diversity is valued and all staff experience a sense of inclusion, featuring equality and non-discrimination [102].

Evidence from this current review indicated insufficient research on SLNs, particularly qualitative, the nature and impact on this group of nurses, and extensive understandings of SLNs. The review also indicates patients and co-workers as perpetrators of WPV towards SLNs. However, it is challenging to ascertain if perpetrators are the same or different compared to the RN group. This scoping review suggests that SLNs' experiences are less understood than RNs, although evidence from one study [57] indicates that SLNs reportedly encounter more physical violence than RNs. Hence, there is insufficient knowledge to draw conclusions. Clearly, more empirical research is needed to provide sufficient information on the experiences of this group of nurses and their support needs.

Regarding educational preparation to combat WPV, two studies explored educational projects on antiviolence programs [51] and WPV safety training programs [58] in the US context. The study by Ridenour et al. [58] reported that RNs (*n* = 203, 78.7%) received a higher proportion of any of the components of WPV training than SLNs (*n* = 97, 56.2%, *p* = 0.0022). Furthermore, the authors found that RNs who attended required training components reported feeling more secure at work, suggesting that training is a critical tool to address WPV [58]. One way to mitigate the magnitude and prevalence of WPV is to include structured educational intervention programs, fostering effective communication skills and using de-escalation techniques in recognising potentially violent patients [103]. For example, Ceravolo et al. [104] proposed a train-the-trainers approach program. Essentially, the aim was to train clinical champions in the workplace to promote expansion of specialist skills and knowledge in reducing WPV by reinforcing nurses' communication skills as an effective and cost-saving approach [104]. Results of the study after employing train-the-trainer workshops series over three years indicated that nurses who reportedly experienced verbal abuse at work reduced from 90% (*n* = 634) to 76% (*n* = 370). In addition, the number of nurses who consider that verbal abuse would influence their overall nursing care delivery reportedly increased from 42% (*n* = 276) to 63% (*n* = 204), indicating strengthened level of awareness [104]. It is imperative that nurse managers and organisations need to prioritise WPV training programs for the nursing workforce, particularly SLNs, so they are well equipped with the knowledge and training they need as frontliners.

Lastly, regarding rates of WPV, the review suggests wide reports of prevalence across the studies, and thus the actual prevalence for this group remains unclear. Similar disparities were recently identified in a systematic review of horizontal violence in novice nurses [105]. Hence, understanding the full scope of WPV for different nursing groups is difficult. The review findings indicated SLNs were less likely to experience electronic abuse compared to FLNs. However, more research is needed to understand the unique WPV experiences of SLNs.

### 4.1. Limitations

Several limitations need to be acknowledged in this current review. Concerning the included literature, the studies were written in English language. It is possible that there may be salient literature written in other languages that may provide more diverse perspectives on the topic. Another limitation is that SLNs are not of particular interest to FLNs. SLNs as a group are poorly researched in comparison to FLNs. Additionally, the included studies are peer-reviewed literature only; the authors did not consult the grey literature during the development of this review; hence, there is a possibility that relevant data might be overlooked that should have been included in the current review. Lastly, despite consultations from the research librarian and trying to capture all names that describe second-level nursing roles in the search strategy, there is a potential that there were missing second-level nursing titles and names that the authors did not identify. Therefore, it could be suggested that the current review is inaccurately delineated and does not accurately represent the SLNs' WPV experience. Regardless of these limitations, this study provided valuable information that can be used to develop further studies on the specific WPV experiences of SLNs.

## 5. Conclusion

This review reports significant insights into the WPV phenomenon experienced by SLNs. As far as the authors are concerned, this is the first review to integrate the published research studies on the SLNs' WPV experiences. The current review provides a comprehensive overview that contributes to new knowledge highlighting the SLNs' WPV experiences worldwide. There are a growing number of studies which are being published and the common themes in the review experienced by SLNs include physical and non-physical violence. However, there is compelling evidence from this review that there are gaps identified and there is a need for greater understandings of WPV in SLNs to understand the scope of their problem and the nature of their experiences. Considering that in the current review, only two studies focused on WPV education programs [51, 58] illuminates the challenges for nurse managers in fostering an inclusive nursing culture. This can be demonstrated by creating a supportive environment and prioritising SLNs' wellbeing, empowering them through valuing ongoing professional development on WPV safety and training program, and improve nurse retention rate by cultivating a respectful, inclusive culture where open communication is encouraged, and recognising and appreciating the nurses' hard work and contributions promote a collaborative team and inclusive culture and establishing a zero-tolerance approach to WPV.

### 5.1. Implications for the Nurse Manager

This review highlights issues of critical importance for nurse managers and organisations on the urgent need to develop and implement effective policies and interventions to improve the working conditions of the SLNs. SLNs are the neglected and understudied group within the nursing profession. Regarding WPV safety training, nurse managers must provide sufficient evidence-based safety training programs to combat WPV. This review also illuminates the need to create an inclusive nursing culture, a culture conducive to a positive working environment where nurses are valued, supported, and provided with utmost protection and security from physical and non-physical violence. Furthermore, this review elucidates the prevalent culture of racial discrimination and sexual harassment. It is crucial that nurse managers are role models where they are proactively committed in creating a diverse and inclusive nursing workforce by strengthening the organisational structure from the top levels of the nursing hierarchy down to the lower levels. The results of this current review can be used to guide nurse managers and hospital organisations in providing adequate support to reduce and prevent WPV, specifically for SLNs.

## Figures and Tables

**Table 1:** Search strategy.

Search (S)	Search terms
S1	“Enrolled Nurs^*∗*^” OR “endorsed enrolled Nurs^*∗*^” OR “second level Nurs^*∗*^” OR “licensed practical Nurs^*∗*^” OR “licensed vocational Nurs^*∗*^” OR “division 2 Nurs^*∗*^” OR “state enrolled Nurs^*∗*^” OR “auxilliary Nurs^*∗*^” OR “nursing auxillary” OR “practical Nurs^*∗*^” OR “associate practical Nurs^*∗*^” OR “registered practical Nurs^*∗*^”

S2	“Workplace violen^*∗*^” OR “horizontal violen^*∗*^” OR “lateral violen^*∗*^” OR “vertical violen^*∗*^” OR “upwards violen” OR “workplace incivility” OR “horizontal incivility” OR “lateral incivility^*∗*^” OR “vertical incivility^*∗*^” OR “workplace aggress^*∗*^” OR “horizontal aggress^*∗*^” OR “lateral aggress^*∗*^” OR “vertical aggress^*∗*^” OR “workplace bull^*∗*^” OR “horizontal bull^*∗*^” OR “lateral bull^*∗*^” OR “vertical bull^*∗*^” OR “workplace conflict” OR “horizontal conflict” OR “lateral conflict” OR “vertical conflict” OR “workplace hostility” OR “horizontal hostility” OR “lateral hostility” OR “vertical hostility” OR “interprofessional violen^*∗*^” OR “horizontal mobbing” OR “lateral mobbing” OR “vertical mobbing” OR “interprofessional conflict” OR “interprofessional bull^*∗*^” OR “interprofessional violen^*∗*^” OR “intra-professional conflict” OR “intra-professional bull^*∗*^” OR “intra-professional conflict” OR “intra-racial conflict” OR “nurse-to-nurse” conflict' OR “nurse-to-nurse aggress^*∗*^” OR “mobbing” OR “horizontal mobbing” OR “incivility” OR “nurses eating their young”

S3	S1 AND S2

**Table 2:** Study characteristics.

Author, year, country	Aim(s)	Study design	Participants, setting	Key findings/results	Study limitations	JBI critical appraisal
Åström et al. (2002) Sweden [45]	(1) To investigate incidence of violence directed towards staff by elderly people living in residential settings and ordinary homes (2) To examine relationships between violent incidents and gather background data regarding staff and their experiences in violent situations	Quantitative using semi-structured, author designed written questionnaires	506 caregiving staff working with the elderly in two settings: residential settings and in the homes of the elderly, of whom 173 were second-level nurses (SLNs) (assistant nurses (ANs)) in two Swedish municipal districts	(1) SLNs reported highest incidence of workplace violence (*n* = 173, 53%) compared to other nurses	Transferability of findings limited due to demographic areas in which the study was undertaken	3/8
				(2) Physical violence the most common type (*n* = 153, 76%) compared with psychological violence (*n* = 25, 12%)		
				(3) Staff working in nursing homes reported highest incidence of violence from residents (*n* = 147, 55%), compared to staff working with the elderly in their homes who reported least violence (*n* = 12, 12%)		
				(4) Male staff reported more exposure to violence (53%) than female staff (38%)		
				(5) Staff involvement in violence reported more by full-time employees (*n* = 114) and staff working in daytime (*n* = 173; 45%, *p* ≤ 0.005 and 45%, *p* ≤ 0.01, respectively), compared with part-time employees (*n* = 87) and those working at night (*n* = 28, 35 and 29%, respectively)		
				(6) Experiences of powerlessness (*n* = 112, 56%), unhappiness (*n* = 102, 51%), and anger were most frequently reported feelings concerning violence		

Boateng and Brown (2022) Canada [46]	To explore experiences of ethnic minority and majority nurses on conflicts faced with patients and patients' family members in direct care practice	Phenomenological study employing one-on-one semi-structured in-depth interviews Data analysed using thematic analysis	66 direct care nurses who identified as ethnic/visible minorities (*n* = 38), and white Canadian nurses (*n* = 28) who worked as registered nurses (RNs) and SLNs (registered practical nurses (RPNs)) from multiple healthcare facility sites of a large cosmopolitan and mid-size health service in two Canadian cities	All participants indicated having experienced at least one form of conflict with a patient or family member of a patient recently during practice	Experiences not generalisable to the entire nursing workforce Difficult to extract SLNs' data The study did not specify the exact number of SLNs	8/10
				Themes: physical assault, verbal aggression		
				17 nurses reported having been physically assaulted by patients		
				Although both whites and ethnic minorities had similar experiences, the latter experienced more verbal assaults		
				Discrimination was most reported by visible minorities. Ethnic minority nurses reported racial slurs, disrespect, and rejection by patients for perceived incompetence		
				Perceived incompetence was reportedly more common with visible minority nurses		
				Six out of the 66 recounted experiences of sexual harassment by patients; five were ethnic minority nurses		

Boström et al. (2012) Canada [47]	To describe frequency of aggressive acts experienced by frontline staff in dementia care: residential Alzheimer's care centres (RACC) and secured dementia units (SDUs) To explore associations between aggressive acts experienced by frontline staff and factors related to the work context and care providers	Cross-sectional survey using validated tools Translating research in elder care (TREC) survey; Alberta context tool (ACT) Maslach burnout inventory (MBI)-general survey (GS) or MBI-GS Workplace violence tool	91 frontline staff working in two models of dementia care: RACC and SDUs, of whom 18 were SLNs (licensed practical nurses (LPNs))	A significant proportion of SLNs were employed in SDUs, compared with RACCs (30% vs. 12%, *p* = 0.03)	Pilot study with small sample size may have decreased power of the statistical analysis Self-reported survey, therefore potential issues of reliability	8/8
				Physical assault (50%)		
				Emotional abuse (48%)		
				SLNs		
				Greater proportion of forced sexual touching and fondling than health care assistants (HCAs) (22.2% vs. 5.5%, *p* = 0.046)		
				SLNs were more likely to make official report to a RN or care manager, while HCAs most likely reported to a SLN or either an SLN and a RN or care manager		

Brophy et al. (2018) Canada [48]	To explore phenomenon of healthcare workers the risks they regularly face of violent physical, sexual, and verbal assaults from patients	Descriptive qualitative study using focus group interviews Data were analysed using thematic analysis	54 representing different healthcare workers from diverse communities, of whom 27 were SLNs (RPNs), working in Ontario, Canada	42.6% reported working or previously working in forensic, psychiatric, emergency, or dementia care units where violent incidents occurred on a regular basis, sometimes as an “everyday” occurrence	Small number of SLNs Findings did not identify risks for violence by specific occupation or type of facility Data saturation not addressed Declaration of potential conflict of interest	9/10
				Inconsistencies in protections provided from one healthcare department to another		
				(1) Clinical risk factors: many incidents occurred in emergency departments, long-term care and geriatric care departments, psychiatric units, detox facilities, and forensic units		
				(2) Environmental risk factors: building design where they worked was often not protective		
				Inadequate safety measures, such as lack of seclusion rooms		
				Areas not protected by alarm systems or the alarms were non-functioning		
				Not having personal alarms, particularly non-nursing staff		
				Nursing stations not having protective shatterproof barriers or adequate egress options		
				(3) Organisational risk factors: working alone or short-staffed, inappropriate staff placement, lack of trained security personnel, healthcare workers' responsibilities during code whites, inadequacy of de-escalation and other training, underreporting, and limited use of restraints		
				Lack of communication between occupational groups regarding potentially violent situations		
				Inconsistent flagging of potentially violent patients		
				(4) Social risk factors: lack of respect for healthcare staff and negative societal attitudes towards women, sexual minorities, and racialised and immigrant workers		
				(5) Economic risk factors: limited resources identified as a contributing factor to violence. Understaffing contributed to patient frustration, boredom, fear, and anger, which could then lead to acting out behaviours		
				Increase in public-private partnership could further erode hospital staffing levels and, in turn, patient care and staff safety		

Eager et al. (2010) Australia [49]	To explore relationships in and between scope of practice and communications among teams of nurses	Descriptive qualitative design using focus groups Data analysed using constant comparison method	30 RNs and SLNs (enrolled nurses (ENs)) in three Sydney metropolitan hospitals (medical/or surgical wards) in New South Wales, Australia	Perceptions from SLNs	Not generalisable Hospital system or working culture was not disclosed Numbers of SLNs unclear	9/10
				(1) Frustration and despair regarding workload and scope of practice		
				(2) Scope of practice issue, conflict, and perceived lack of respect for SLN experience and skills		
				(3) SLNs discussed workloads and teamwork as interchangeable realities in their day-to-day work. Perceived inequities in distribution of workloads expressed as “us and them” phenomenon between RNs and SLNs		
				(4) Issue of scope of practice and confusion		
				(5) Some SLNs critical of pay disparity between SLNs and RNs perceiving no difference between roles		
				Expectation added responsibility of medication distribution would relieve endorsed SLN of some traditional work and not add to it		
				(6) SLNs reported feeling ignored, belittled, disposable, and complained of being denied access to handover by RNs in their nursing team		
				(7) SLNs' ability to fully utilise their advanced practical skills (medication administration) did not only depend on the ward they worked in, but also the RN they were working with		
				(8) Role and practice of SLNs shaped not by policy and procedure but by whom the SLN was working with, their years of experience, and sometimes by understaffing issues		
				(9) Some SLNs found scope of practice, conflict issues, and work environment problematic		

Eastman (2013) Canada [50]	To contribute to victim precipitation theory by examining the predictive value of the state-like characteristic of psychological capital (hope, optimism, resiliency, and efficacy) and being a target of workplace bullying	Cross-sectional using online survey with validated tools Psychological capital questionnaire (PCQ) Negative acts questionnaire-revised (NAQ-R)	206 SLNs (practical licensed nurses (PLNs)) from the College of Practical Licensed Nurses of Alberta (CPLNA), Alberta, Canada	41.3% self-labelled as being bullied, and 125 (60.7%) reported at least one bullying behaviour	Only 10 males participating gender excluded as a control variable SLNs from one province selected to participate	8/8
				SLNs had witnessed or been bullying targets		
				The state-like characteristics of psychological capital: hope, optimism, resiliency, and efficacy were related to being a target of workplace bullying		
				Psychological capital provided mild protection against being a target of workplace bullying		

Embree et al. (2013) USA [51]	To determine perceived extent and increase awareness of nurse-to-nurse lateral violence (NNLV) through an educational project about NNLV and cognitive rehearsal (CR)	Quasi-experimental pre- and post-survey using: Nurse workplace behaviour scale (NWS) and silencing the self-work scale (STTS-W) Focus group	135 critical access hospitals (CAHs, or hospitals with 25 or fewer inpatient beds)	No bullying behaviours (never or now and then) reported by 81 (39.3%) SLNs, and 125 (60.7%) reported at least one bullying behaviour (monthly, weekly, or daily) Frequency of bullying behaviours reported ranged from one (*n* = 22, 10.7%) to 20 behaviours (*n* = 2, 1%) Highest rated behaviours (weekly or daily) included being exposed to unmanageable workload (*n* = 54, 26.2%), being ordered to work below level of confidence (belief in own capability; *n* = 47, 22.8%), withholding information which affected performance (*n* = 45, 21.8%), having opinions and views ignored (*n* = 18, 20.9%), and having responsibility removed (*n* = 14, 19.9%) Lowest rated behaviours included practical jokes carried out by people they did not get along with (*n* = 5, 2.4%), threats of violence or physical abuse (*n* = 6, 2.9%), being subject of excessive teasing hints (*n*–11, 5.3%), and having allegations made against them (*n* = 13, 6.3%) Scale	One CAH hospital so findings not generalisable Project director was chief clinical officer/nurse executive in CAH Small sample size of SLNs and difficult to identify data from SLNs	5/9
			Pre-survey: 48 (35%) of 135 CAH nurses participated			
			Post-survey: 35 (24%) of the 143 nurses participated were focus group participants for the pre-survey (*n* = 8)			
			Educational preparation			
			Pre-survey (*n* = 44); 9% SLNs (LPNs)			
			Post-survey (*n* = 33); 3% SLNs,			
			Focus group (*n* = 8), 24% SLNs			

Ericksen et al. (2006) Norway [52]	To identify physical, psychological, social, and organisational work factors that predict level of psychological distress in nurses' aides	Prospective cohort study using questionnaire based on validated tools Symptom checklist-25 (SCL-5) that measures psychological distress and general Nordic questionnaire for psychological and social factors at work (QPSNordic)-measure of working conditions, and measures of mastery and commitment	5076 members of Norwegian's union of health and social workers, the Union's, of whom 4076 were SLNs (nurses' aides (NAs)) who completed second questionnaire and answered at least three questions about psychological distress 15 months later	Exposure to role conflicts, exposure to threats and violence, working in apartment units for the aged, and changes in the work situation between baseline and follow-up that were reported to result in less support and encouragement were positively associated with the level of psychological distress. Working in psychiatric departments and changes in the work situation between baseline and follow-up that gave lower work pace were negatively associated with psychological distress	Response rate at baseline not optimal	10/11
					Potential selection bias	
					Internal consistency index of control over workplace was relatively low	
					Changes in work situation between baseline and follow-up also represent uncertainty in assessment of work factors	

Filipova (2018) USA [53]	To investigate association of authentic leadership (AL) and perceived organisational support (POS) to workplace bullying among licensed practical nurses	Cross-sectional survey using validated tools (1) Authentic leadership questionnaire (ALQ) (2) Negative acts questionnaire-revised (NAQ-R) Survey of perceived organisational support (SPOS)	168 SLNs (LPNs) in Midwest, USA	72 (43%) SLNs experienced at least two negative acts on a weekly/daily basis in previous six months	(1) Cannot infer causality (2) Self-reported data can lead to common method variance and issues of reliability (3) Data collected from SLNs minimised (4) Low response rate limits generalisability of findings	6/8
				Most frequently reported negative acts on a weekly/daily basis: withholding information that affected performance (32% (95% CI, 29%–35%))		
				Having opinions ignored (36% (95% CI, 33%–39%)) and being exposed to unmanageable workload, 34% (95% CI, 31%–37%)		
				Only 2% of nurses experienced extreme negative acts (threat of violence, physical abuse) on weekly/daily basis		
				Of the 42% (95% CI, 39%–45%) who self-identified as bullied, 12% (95% CI, 9%–15%) reported being bullied weekly/daily		
				Most common perpetrators were peers (23%), followed by immediate supervisors (18%) and other supervisors (17%)		
				Work-related bullying was more prevalent (mean, 2.51 [SD, 1.05]) than person-related bullying (mean, 1.8 [SD, 0.96]) and physical intimidation (mean, 1.44 [SD, 0.70])		

Gerberich et al. (2004) USA [54]	To identify magnitude of and potential risk factors for violence within a major occupational population	Case-control study using Minnesota nurses' survey in two phases	4918 Minnesota licensed nurses, of whom were SLNs (LPNs *n* = 950), USA	711 physical assault events reported by 476 nurses who completed full surveys; one, two, three, four, or “ongoing” events were reported by 280, 81, 29, 32, and 54 nurses	Participants self-reported violence and relevant exposures	10/10
				Assault rate of 13.2 per 100 persons per year		
				Adjusted rates for SLNs (16.4), compared to RNs (12.0), differed by a similar amount from unadjusted		
				Adjusted overall non-physical violence rate of 38.8 was slightly higher than the unadjusted rate		
				For SLNs, adjusted rates were 39.7, and RNs were 38.5, respectively, differing slightly from unadjusted rates		

Kennedy and Hester (2013) South Africa [55]	To describe patients' perceptions of possible environmental and staff factors contributing to their aggressive and violent behaviour after admission to a mental health facility To propose strategies to prevent and manage such behaviours	Qualitative exploratory study using semi-structured interviews and observational notes Data analysed using thematic analysis	8 trauma and emergency department nurses at a large academic hospital, Western Cape, South Africa, of whom were SLNs (ENs *n* = 2)	Themes (1) Interpretation of workplace violence: verbal abuse, physical violence, psychological violence, imminent violence	Limited to workplace violence perpetrated only by patients against nurses Small SLN population. Data saturation was not addressed with respect to the small sample	7/10
				(2) Experiences and responses to violence: normalising abusive patient behaviour, under-reporting		
				(3) Coping strategies: institutional support network, external social support network		
				(4) Effects on work performance: compromised care		

MacLeod et al. (2022) Canada [56]	To explore characteristics and context of practice of registered nurses (RNs), licensed practical nurses (LPNs), and registered psychiatric nurses (RPNs) in rural and remote Canada who provide care to those experiencing mental health concerns	Cross-sectional survey using Job resources in nursing (JRDIN) scale and job demands in nursing (JDIN) scale	3,457 nurses residing in rural and remote Canada (RRNII), of whom 1,313 were SLNs (LPNs) working in a full range of practice settings (primary care, acute care, community health, home care, mental health and addictions, and long-term care)	In mental health only practice, more than 50%, especially SLNs, experienced or witnessed violence in the previous 4-week period	Cross-sectional in nature Rural, remote characteristics, context, challenges for nurses who provide mental health nursing	6/8
				(1). Mental health plus SLNs had lowest perceived work-related resources		
				(2) Mental health only SLNs had highest incidences of experiencing and witnessing violence within the previous four weeks		
				Patient/client was most common instigator of violence experienced by nurses in the mental health only group, mental health plus nurses, and most types of witnessed violence for the mental health only nurses. Similar proportions were noted for mental health plus nurses, with patient/client as most common perpetrator of witnessed violence		

Nachreiner et al. (2007) USA [57]	To compare experiences of work-related violence among registered nurses and licensed practical nurses to quantify differences in risks and exposure and to gain insight into possible interventions	Case-control study using Minnesota nurses' survey in two phases (reports on phase 1 whether respondents worked as nurses in Minnesota in the previous 12 months and ascertained descriptions of any work-related violence experienced during that time)	4918 Minnesota nurses, of whom were SLNs (LPNs *n* = 950), Minnesota, USA	Adjustment for potential response bias resulted in physical violence rates for SLNs 16.4 per 100 nurses per year. Adjusted non-physical violence rates for SLNs were 39.7 per 100 nurses	Participants self-reported violence and relevant exposures	8/8
				SLNs more likely than RNs to be assaulted (odds ratio (OR) = 1.4; 95% confidence interval (95% CI) = 1.1–1.9). Non-physical violence more likely for SLNs than RNs (OR = 1.2; 95% CI = 1.0–1.5)		
				Sexual harassment reported by 7% SLNs, threats reported by 17% of SLNs, and verbal abuse reported by 36% of SLNs		
				Majority of physical violence events perpetrated by patients/clients for SLNs (97%)		
				Perpetrators of non-physical violence included patients/clients for 78% SLNs		
				Majority of perpetrators of physical violence perceived to be impaired because of disease/illness or prescribed medication (90% SLNs), male (61% SLNs), and aged 66 years (74% LPNs). Perpetrators associated with non-physical violence perceived as non-impaired (38% SLNs) and primarily male (77% SLNs). A greater proportion of non-physical violence perpetrators were younger (35–65 years; 41% SLNs)		
				Physical assaults occurred primarily in patient rooms and hallways for both RNs and SLNs		
				Non-physical violence events occurred primarily face-to-face (91% SLNs) or telephone (11% SLNs)		
				Most denied receiving treatment for injuries resulting from physical violence (74% SLNs); those indicating some treatment often reported self-care (20% SLNs)		
				Greater percentage of nurses reported treatment following non-physical violence (9% SLNs for both SLNs and RNs) than following physical violence (5% for both SLNs and RNs)		
				Persistent problems resulting from the event were reported by 6% of SLNs experiencing physical violence, 10% of SLNs reporting persistent problems resulting from non-physical violence		
				11% SLNs had job changes following physical violence event, compared with 20% of RNs and SLNs following a non-physical violence event		
				76% of SLNs reported events to management, verbally, in writing, or both		
				SLNs were more likely to report non-physical violence to management than were RNs, with 80% of SLNs reporting events compared to 68% of RNs		
				Increased risk of physical assault for SLNs working primarily with geriatric patients or in psychiatric/behavioural departments and working in nursing home/long-term care facilities		
				Decreased risks for SLNs working primarily in clinics/healthcare provider offices		
				SLNs had an increased risk of physical assault when supervising patient care; working with neonatal/paediatric patients; and having worked in their department for ≥10 years		
				SLNs had increased risk of non-physical violence when they graduated from nursing school between 1980 and 1989 (compared with graduation before 1970); however, SLNs had decreased risks of non-physical violence when they worked in public health/education/school/research departments; were female; and had baccalaureate degrees or higher for their most advanced nursing educational credential		

Ridenour et al. (2017) USA [58]	To examine nurses' knowledge of the state of New Jersey violence prevention in health care facilities act, workplace violence training, and experience with workplace violence	Cross-sectional study using survey	309 nursing staff, of whom were SLNs (LPNs *n* = 97)	RNs (78.7%) received higher proportion of training than SLNs (56.2%) (*P* = 0.0022)	Recall and reporting biases since participants self-reported violence events and training they received Better response rate from RNs than SLNs	
				Respondents working in hospital (77.8%) received a higher proportion of training than those working in nursing homes (62.3%) (*P* = 0.0122)		
				For those who received 80% training, the following were significant: day shift (35.3%) vs. evening shift (46.3%) (*P* = 0.0242), female (35.6%) vs. male (53.4%) (*P* = 0.0419), and always secure at work (51.1%) vs. mostly secure at work (30.9%) vs. some/rarely/never secure at work (35.8%) (*P* = 0.0043)		
				Verbal and physical abuse were the most common events when perpetrator was a patient/family member		
				When perpetrator was a co-worker/administrator, most frequently experienced violent events SLNs were bullying, verbal abuse, and threats		
				Higher proportion of threats experienced on rotating shifts than on day and evening/night shifts		
				Potential reasons why SLNs received less training: higher turnover, lesser status in hierarchy of employees, or working a night shift		
				Nurses who received at least 80% of required training components were more likely to feel more secure		

Shea et al. (2017) Australia [59]	To examine extent and source of occupational violence and aggression (OVA) experienced by nursing and caring professionals To examine relative contributions of demographic characteristics and workplace and individual safety factors in predicting OVA	Cross-sectional study using occupational health and safety (OHS) online survey data	4,891 members of Australian Nursing and Midwifery Federation (ANMF, Victorian branch), of whom 1,055 were SLNs (ENs)	67% reported experiencing OVA at least once in the previous 12 months, and nearly 20% reported experiencing on a weekly/daily basis	Low-response rate may indicate sampling bias	7/8
				SLNs and personal carers (PCs) (71%, 76%, respectively) showed higher prevalence of OVA compared to RNs and midwives (66%, 63%, respectively), but findings were not statistically significant		
				SLNs (RNs and PCs) were more likely to experience OVA from patients rather than patient relatives or visitors, compared to midwives who were more likely to experience OVA from patient relatives and visitors than patients themselves		

Small et al. (2015) USA [60]	To determine incidence of disruptive behaviour among nurses in the health workplace, details associated with its occurrence, and organisational procedures utilised when disruptive incidents occur	Cross-sectional study using online survey	2,795 Florida board of nursing licensed nurses, of whom were SLNs (LPNs *n* = 275), USA	SLNs and RNs (87.9%, 86.8%, respectively) reported being the victims of verbal abuse, compared to advanced practiced registered nurses (APRNs, 73.2%)	Potential self-reporting bias	6/8
				SLNs and RNs (18.8%, 22.8%, respectively) reported the most instances of physical abuse, compared to ARNPs (10.9%)		
				Most physical abuse cases indicated client/patient was the abuser (56.7%), compared to 21.4% of abusers who were co-workers		
				SLNs (7.1%) reported being least victims of e-mail abuse, compared to nursing positions: “other,” ARNP and RNs (18.3%, 13.2%, 11.5%, respectively)		
				SLNs were the most likely to feel the organisation was not protected		
				SLNs were more likely to miss work due to abuse (than APRNs)		

Tong et al. (2017) Switzerland [61]	To examine frequency of mobbing in Swiss nursing homes and its relationships with care workers' health status, job satisfaction, and intention to leave, and to explore the work environment as a contributing factor to mobbing	Cross-sectional using negative acts questionnaire (short version); practice environment scale-nursing work index (PES-NWI); safety climate and teamwork of the safety attitudes questionnaires (SAQ); Michigan organisational assessment questionnaire (MOAQ)	5311 care workers in Swiss nursing homes, of whom 1,171 were SLNs (LPNs)	242 (4.6%) reported experiencing mobbing in the workplace	Cross-sectional design so causal relationships cannot be inferred	8/8
				Prevalence was lower among nurse aides (*n* = 36, 2.6%) and other professionals (*n* = 3, 1.8%) compared to RNs (*n* = 94, 5.9%), SLNs (*n* = 62, 5.0%), and certified assistant nurses (*n* = 36, 5.0%). The prevalence of workplace mobbing was higher among SLNs (and RNs) compared to nurse aides and other professionals		

Welch et al. (2013) USA [62]	To ascertain whether veterans health administration (VHA) administrative medical centre complexity ratings could be used to help identify potential sites for targeted nursing staff workplace violence intervention activities	Retrospective prevalence study analysing incident report outcome measures	1,854 field-based VHA nursing staff, of whom 62 were SLNs (practical nurses (PNs))	As of September 30, 2011 (fiscal years 2004–2011), a total of 9,964 assault incidents reported: SLNs (2,039, or 10.6% of all reported incidents), compared to nurses (3,580, or 7.2%)	Only included reported incidents so actual may have been higher	9/9
				For female staff, average per annum reported assault rate (per 10,000 employees) was 237.8 for SLNs		
				Average per annum assault rates (per 10,000 employees) for male staff were 254.4 for SLNs. Across all fiscal years combined, standardised incidence rates for reported assaults among females were 2.3 times higher for SLNs		
				For males, corresponding rates for SLNs were 1.5 times higher		

**Table 3:** Specific aspects of SLNs' WPV experiences.

Author, year	Nature of violence/conflict	Long-term impact/outcome	Prevention and coping strategies
Åström et al. (2002) [45]	(i) Physical	(i) Powerlessness and sadness (ii) Anger and feelings of insufficiency Guilt and shame	Not stated
	(ii) Psychological		
	(iii) Economic Sexual		

Boateng and Brown (2022) [46]	(i) Physical assault (ii) Verbal aggression (iii) Sexual harassment (iv) Family demands and abuse (v) Racial discrimination	(i) Implications for absenteeism and turnover (ii) Feeling of vulnerability (iii) White nurses are perceived as superior and competent, and ethnic minority nurses as inferior and less incompetent	Nurses' coping mechanisms
			(i) When the target of physical abuse, nurses called for hospital security
			(ii) Switched with other nurses who had better work relationships with the patient
			(iii) Debriefing with co-workers whom they found to understand their situation
			(iv) Venting to family members/close friends upon arriving at home
			(v) Working in pairs, especially with patients flagged as uncivil
			(vi) Following hospital protocols specifically developed for dealing with violent cases
			(vii) Seeking help from colleagues and devoting time to mitigating such situations
			(viii) Talking with patients and family members about their behaviour to ease stress family members were under
			(ix) Ethnic minority nurses stressed venting to nurses of similar ethnic identity for social support and networking
			(x) Some ethnic minority nurses were conscious in providing excellent care to patients while some destress by resorting to religious activities including church attendance and prayers
			Institutional support
			(i) Policies and practices designed to address conflict
			(ii) Some hospitals discharged/dismissed patients for abusive behaviour; this mode of resolving conflicts was reported more often by participants

Boström et al. (2012) [47]	(i) Physical (ii) Emotional (iii) Verbal (iv) Sexual harassment	(i) Lack of supervisors' awareness and management of WPV aggression, and inappropriate models of care (ii) Organisational slack: staffing, space, and time	(i) Strategies aiming to detect or prevent aggressive behaviour at an early age
			(ii) Informal individual communication with colleagues to manage violent incidents
			(iii) Strategies aimed at calming and de-escalating when signs of aggressive behaviour appear
			(iv) Repressive or sanctioning interventions
			(v) Medical treatment
			(vi) Policy-based strategies

Brophy et al. (2018) [48]	(i) Physical (ii) Psychological (iii) Interpersonal	(i) Violence becomes “normalised” or “part of the job” in nursing, and impossible to eliminate the threat of violence (ii) Sense of hopelessness and defeat (iii) Failure to recognise/compensate for many psychological and physical effects of violence leading to serious conditions (PTSD, cumulative stress, and long-term effects of concussion) (iv) Inconsistency of budgets, chronic underfunding of healthcare system leading to failure of implementing protections (adequate staffing, programs, engineering controls, environmental design, security)	Primary prevention
			(i) Increase staffing
			(ii) Enhanced security
			(iii) Flag violent patients
			(iv) Personal alarms
			(v) Building design changes
			(vi) Zero tolerance policies
			(vii) Better training and flagging
			Secondary prevention
			(i) Encourage incident reporting
			(ii) Simplified reporting process
			(iii) Using the criminal justice system
			Tertiary prevention
			(i) Better training and flagging

Eastman. (2013) [50]	(i) Bullying	(i) Stress	(i) Hope (ii) Optimism (iii) Resiliency (iv) Efficacy
		(ii) Suicidal ideation	
		(iii) PTSD symptoms	
		(iv) Increased medication errors	
		(v) Late delivery of medication administration	
		(vi) Consider leaving the occupation	

Embree et al. (2013) [51]	(i) Bullying	(i) Not stated	(i) Nurses encouraged to share stories of NNLV with other nurses to assist in decreasing the NNLV behaviour by describing effective methods of deflecting this behaviour

Erikssen et al. (2006) [52]	(i) Exposure to role conflicts and threats	Not stated	Not stated
	(ii) Violence at work		

Eager et al. (2010) [49]	(i) Scope of practice (ii) Teamwork (iii) Team conflict	(i) Continued disunity and conflict among RNs and ENs	“Healthy” coping mechanisms
		(ii) Territorial conflicts affect staff and quality of patient care	(i) Consulting colleagues, humour, and seeking advice from their managers
		(iii) Division of labour and responsibilities leads to miscommunication, bullying, and harassment	Radical mechanisms (less satisfactory or long-term solutions):
		(iv) Some RNs have lack of insight, the negative impact of their behaviour to their co-workers	(i) Leaving the ward, manipulating the nursing staff roster to avoid working the same shifts with the other staff, or “shutting down to avoid further conflicts”

Filipova (2018) [53]	(i) Overall workplace bullying	(i) Developing nurses' authentic leadership (AL) capabilities	(i) Supportive environment
	(ii) Person-related bullying		(ii) Encourage employee beliefs that the organisation is committed to antibullying policies and processes
	(iii) Physical intimidation		(iii) Teamwork, open communication, and genuine relationships—staff willingness to voice concerns about bullying

Gerberich et al. (2004) [54]	(i) Physical (ii) Non-physical	(i) Frustration	(i) Flagging the chart of a violent patient (ii) Assigning two caregivers and using appropriate strategies for a combative patient
		(ii) Anger, fear/anxiety/stress	
		(iii) Irritability	
		(iv) Fatigue	
		(v) Sadness	
		(vi) Headaches	
		(vii) Difficulty concentrating	
		(viii) Sleeplessness	
		(ix) Shame/low self-esteem	
		(x) Depression	
		(xi) Flashbacks	
		(xii) Reduced productivity, increased turnover, absenteeism, decreased staff morale	
		(xiii) Counselling costs	
		(xiv) Reduced quality of life	
		(xv) Physical injury, disability	
		(xvi) Changes in job performance and morale	
		(xvii) Chronic pain, muscle tension	
		(xviii) Restrictions/modified work	

Kennedy and Hester (2013) [55]	(i) Physical violence (ii) Verbal abuse (iii) Psychological violence (iv) Imminent violence	(i) Normalising abusive patient behaviour (ii) Underreporting of violence	Institutional support network:
			(i) Using colleagues—helping out with duties, taking a cigarette break
			(ii) Using friends and family—to get it “off their chest”
			(iii) Physical support—peers protect against the abuse or take over the nursing care of the violent or abusive patient
			(iv) Emotional support provides immediate relief, validation, and empathic understandings from colleagues
			External social support network:
			(i) Family and friends play an essential role by providing encouragement to deal with WPV
			(ii) Supportive counselling for individuals/peer groups

MacLeod et al. (2022) [56]	(i) Physical (ii) Non-physical	(i) Gaps in services	Not stated
		(ii) Inadequate security protocols and supports and limited backup for nurses in situations of violence	
		(iii) Impact staff retention and exacerbate long-term nursing shortages	
		(iv) Perpetuate inequitable provision of care for those with mental health concerns in rural and remote communities	
		(v) Limited housing and transportation options placed greater demands to find innovative solutions	
		(vi) Inadequate resources for training, professional development, and continuing education in de-escalating violence and dealing with unsafe situations	
		(vii) Withdrawal from community engagement	
		(viii) Intent to leave the profession	
		(ix) Exacerbate long-term nurse shortage and retention	

Nachreiner et al. (2007) [57]	(i) Physical violence (ii) Non-physical violence	(i) Frustration, anger	Not stated
		(ii) Fear, anxiety, stress, irritability	
		(iii) Fatigue, difficulty sleeping, difficulty concentrating	
		(iv) Sadness	
		(v) Shame/low self-esteem	
		(vi) Depression	
		(vii) Headache	
		(viii) Flashbacks, nightmares, hallucinations	
		(ix) Changes in job status	

Ridenour et al. (2017) [58]	(i) Physical assault	Not stated	(i) Workplace violence training
	(ii) Verbal abuse		
	(iii) Threats		

Shea et al. (2017) [59]	(i) Role overload	Not stated	(i) Safety compliance
	(ii) Individual safety factors		(ii) Safety motivation
	(iii) Workplace safety factors		(iii) Safety participation

Small et al. (2015) [60]	(i) Physical abuse	Not stated	(i) Felt comfortable reporting WPV incidents to supervisor, manager, or someone else
	(ii) Verbal abuse		
	(iii) Electronic abuse		

Tong et al. (2017) [61]	(i) Mobbing	(i) Attitudinal: job satisfaction, intention to leave	(i) Effective leadership
		(ii) Behavioural: absenteeism, presenteeism	(ii) Staffing and resource adequacy
		(iii) Health issues: neck and back pains—fatigue, tiredness, lack of energy; problems with falling asleep or staying asleep; headaches, feelings of pressure in the head, or facial pains; aching limbs or joints	(iii) Teamwork and safety climate

Welch et al. (2013) [62]	(i) Occupational assault incidents	(i) Incidence rates for reported assaults for 2 groups of body parts: arms and hands, head and neck	Not stated
		(ii) Financial impacts: lost employee work hours when violence results in psychological or physical injury	
		(iii) Salary replacement dollars for overtime/agency staff	
		(iv) Potential legal ramifications from lawsuits	
		(v) Negative publicity impacts on recruitment and retention	
		(vi) Patient care impacts	
		(vii) Significant impact on delivery of quality of care, increased documentation and medical errors, and decreased attention to patient care needs,	

## Data Availability

No data were used to support this study.

## Supplementary Materials

Supplementary Materials PRISMA Checklist Guideline [36].

## References

[1] International Labour Organization (2019). Eliminating violence and harassment in the world of work. *Convention No. 190, Recommendation No. 206, and The Accompanying Resolution*.

[2] Spelten E., Thomas B., O’Meara P. F., Maguire B. J., Fitzgerald D., Begg S. J. (2017). Organisational interventions for preventing and minimising aggression directed toward healthcare workers by patients and patient advocates. *Cochrane Database of Systematic Reviews*.

[3] Phillips J. P. (2016). Workplace violence against health care workers in the United States. *New England Journal of Medicine*.

[4] Fang H., Zhao X., Yang H. (2018). Depressive symptoms and workplace-violence-related risk factors among otorhinolaryngology nurses and physicians in Northern China: a cross-sectional study. *BMJ Open*.

[5] Zhao S., Xie F., Wang J. (2018). Prevalence of workplace violence against Chinese nurses and its association with mental health: a cross-sectional survey. *Archives of Psychiatric Nursing*.

[6] Magnavita N., Di Stasio E., Capitanelli I., Lops E. A., Chirico F., Garbarino S. (2019). Sleep problems and workplace violence: a systematic review and meta-analysis. *Frontiers in Neuroscience*.

[7] Itzhaki M., Bluvstein I., Peles Bortz A. (2018). Mental health nurse’s exposure to workplace violence leads to job stress, which leads to reduced professional quality of life. *Frontiers in Psychiatry*.

[8] Gates D. M., Gillespie G. L., Succop P. (2011). Violence against nurses and its impact on stress and productivity. *Nursing economic$*.

[9] Zhao S. H., Shi Y., Sun Z. N. (2018). Impact of workplace violence against nurses’ thriving at work, job satisfaction and turnover intention: a cross-sectional study. *Journal of Clinical Nursing*.

[10] Lipscomb J. A., El Ghaziri M. (2013). Workplace violence prevention: improving front-line health-care worker and patient safety. *New Solutions: A Journal of Environmental and Occupational Health Policy*.

[11] Speroni K. G., Fitch T., Dawson E., Dugan L., Atherton M. (2014). Incidence and cost of nurse workplace violence perpetrated by hospital patients or patient visitors. *Journal of Emergency Nursing*.

[12] United States Department of Labour (2022). Business case for safety and health. Safety and health topics. https://www.osha.gov/businesscase.

[13] Pich J., Roche M. (2020). Violence on the job: the experiences of nurses and midwives with violence from patients and their friends and relatives. *Healthcare*.

[14] Nowrouzi-Kia B., Isidro R., Chai E., Usuba K., Chen A. (2019). Antecedent factors in different types of workplace violence against nurses: a systematic review. *Aggression and Violent Behavior*.

[15] Dafny H. A., Muller A. (2021). Australian nurses’ suggestions for the management of violence in the workplace: ’The people who make the policy are not the people on the floor. *Journal of Nursing Management*.

[16] Spelten E., Thomas B., O’Meara P., van Vuuren J., McGillion A. (2020). Violence against emergency department nurses: can we identify the perpetrators?. *PLoS One*.

[17] International Council of Nurses (ICN) (2022). International nurses day (ind), nurses: a voice to lead- invest in nursing and respect rights to secure global health. https://www.icn.ch/news/greatest-threat-global-health-workforce-shortage-international-council-nurses-international.

[18] England Health Education (2017). *Facing the Facts, Shaping the Future: A Draft Health and Care Workforce Strategy for England to 2027*.

[19] Schwartz S. (2019). *Educating the Nurse of the Future- Report of the Independent into Nursing Education*.

[20] Butcher D. L., MacKinnon K. (2015). Educational silos in nursing education: a critical review of practical nurse education in Canada. *Nursing Inquiry*.

[21] Chua W. L., Mackey S., Ng E. K. C., Liaw S. Y. (2013). Front line nurses’ experiences with deteriorating ward patients: a qualitative study. *International Nursing Review*.

[22] Chan Z. C. Y., Lai W. (2010). A Hong Kong perspective on ways to improve nurse retention. *Nursing Standard*.

[23] Lucas G., Daniel D., Thomas T., Brook J., Brown J., Salmon D. (2021). Healthcare professionals’ perspectives on enrolled nurses, practical nurses and other second-level nursing roles: a systematic review and thematic synthesis. *International Journal of Nursing Studies*.

[24] Clayton-Hathway K., Laure Humbert A., Griffiths H., Mcilroy R., Schutz S. (2020). *Gender and Nursing as a Profession: Valuing Nurses and Paying Them Their worth*.

[25] Roberts S. J. (1983). Oppressed group behavior: implications for nursing. *Advances in Nursing Science*.

[26] Chakraborty B., Mandal A., K Sharma S. (2021). Are Nurses in Oppression? An approach to explore the evidences. *Saudi Journal of Nursing and Health Care*.

[27] Matheson L. K., Bobay K. (2007). Validation of oppressed group behaviors in nursing. *Journal of Professional Nursing*.

[28] Blay N., Smith L. E. (2020). An integrative review of Enrolled Nurse recruitment and retention. *Collegian*.

[29] Currie J., Grootemaat P., Samsa P., Halcomb E., Thompson C. (2019). *Topic 3: Clinical Skill Development*.

[30] Leon R. J., Tredoux J. H., Foster S. M. (2019). Valuing Enrolled Nurses – a study to better understand the investment education and training have on the retention of Enrolled Nurses. *Collegian*.

[31] Phillips L. A., De Los Santos N., Jackson J. (2021). Licenced practical nurses’ perceptions of their work environments and their intention to stay: a cross‐sectional study of four practice settings. *Nursing Open*.

[32] Chua W. L., Legido-Quigley H., Ng P. Y., McKenna L., Hassan N. B., Liaw S. Y. (2019). Seeing the whole picture in enrolled and registered nurses’ experiences in recognizing clinical deterioration in general ward patients: a qualitative study. *International Journal of Nursing Studies*.

[33] Colquhoun H. L., Levac D., O’Brien K. K. (2014). Scoping reviews: time for clarity in definition, methods, and reporting. *Journal of Clinical Epidemiology*.

[34] Arksey H., O’Malley L. (2005). Scoping studies: towards a methodological framework. *International Journal of Social Research Methodology*.

[35] Levac D., Colquhoun H., O’Brien K. K. (2010). Scoping studies: advancing the methodology. *Implementation Science*.

[36] Page M. J., Mckenzie J. E., Bossuyt P. M. (2021). The PRISMA 2020 statement: an updated guideline for reporting systematic reviews. *BMJ*.

[37] Australian Nursing and Midwifery Council (2002). *National Competency Standards for the Enrolled Nurse, Australian Nursing and Midwifery*.

[38] Gora Y. (2017). Regulations of nursing associates in england. A paper for consultation on amendments to the nursing and Midwifery order 2001 and subordinate legislation to regulate nursing associates in england by the nursing and Midwifery Council. https://assets.publishing.service.gov.uk/government/uploads/system/uploads/attachment_data/file/652658/Rona-consultation.pdf.

[39] La Trobe University (2022). Covidence and screening records-Systematic Reviews. https://latrobe.libguides.com/systematicreviews.

[40] Joanna Briggs Institute (JBI) (2017). Critical appraisal tools. https://joannabriggs.org/research/critical-appraisal-tools.

[41] Cooper S., Cant R., Kelly M. (2021). An evidence-based checklist for improving scoping review quality. *Clinical Nursing Research*.

[42] Frambach J. M., van der Vleuten C. P. M., Durning S. J. (2013). AM last page: quality criteria in qualitative and quantitative research. *Academic Medicine: Journal of the Association of American Medical Colleges*.

[43] Munn Z., Peters M. D. J., Stern C., Tufanaru C., Mcarthur A., Aromataris E. (2018). Systematic review or scoping review? Guidance for authors when choosing between a systematic or scoping review approach. *BMC Medical Research Methodology*.

[44] McKenzie J. E., Brennan S. E., Ryan R. E., Thomson H. J., Johnson R. V., Higgins J. P. T., Thomas J., Chandler J. (2019). Summarizing study characteristics and preparing for synthesis. *Cochrane Handbook for Systematic Reviews of Interventions*.

[45] Åström S., Bucht G., Eisemann M., Norberg A., Saveman B.-I. (2002). Incidence of Violence towards staff caring for the elderly. *Scandinavian Journal of Caring Sciences*.

[46] Boateng G. O., Brown K. K. (2022). Go back to your country: exploring nurses’ experiences of workplace conflict involving patients and patients’ family members in two Canadian cities. *Nursing Inquiry*.

[47] Boström A.-M., Squires J. E., Mitchell A., Sales A. E., Estabrooks C. A. (2012). Workplace aggression experienced by frontline staff in dementia care. *Journal of Clinical Nursing*.

[48] Brophy J. T., Keith M. M., Hurley M. (2018). Assaulted and unheard: violence against healthcare staff. *New Solutions: A Journal of Environmental and Occupational Health Policy*.

[49] Eager S. C. E., Cowin L. S., Gregory L., Firtko A. (2010). Scope of practice conflict in nursing: a new war or just the same battle?. *Contemporary Nurse: A Journal for the Australian Nursing Profession*.

[50] Eastman G. B. (2013). *The Relationship between Psychological Capital and Workplace Bullying for Nurses*.

[51] Embree J. L., Bruner D. A., White A. (2013). Raising the level of awareness of nurse-to-nurse lateral violence in a critical access hospital. *Nursing Research and Practice*.

[52] Eriksen W., Tambs K., Knardahl S. (2006). Work factors and psychological distress in nurses’ aides: a prospective cohort study. *BMC Public Health*.

[53] Filipova A. A. (2018). Countering unprofessional behaviors among nurses in the workplace. *The Journal of Nursing Administration: The Journal of Nursing Administration*.

[54] Gerberich S. G., Church T. R., McGovern P. M. (2004). An epidemiological study of the magnitude and consequences of work-related Violence: the Minnesota Nurses’ Study. *Occupational and Environmental Medicine*.

[55] Kennedy M., Hester J. (2013). Nurses’ experiences and understanding of workplace violence in a trauma and emergency department in South Africa. *Health SA Gesondheid: Journal of Interdisciplinary Health Sciences*.

[56] MacLeod M., Penz K. L., Banner D. (2022). Mental health nursing practice in rural and remote Canada: insights from a national survey. *International Journal of Mental Health Nursing*.

[57] Nachreiner N. M., Hansen H. E., Okano A. (2007). Difference in work-related Violence by nurse license type. *Journal of Professional Nursing: Official Journal of the American Association of Colleges of Nursing*.

[58] Ridenour M. L., Hendricks S., Hartley D., Blando J. D. (2017). Workplace violence and training required by new legislation among NJ nurses. *Journal of Occupational and Environmental Medicine*.

[59] Shea T., Sheehan T., Donohue R., Cooper B., Cieri H. (2017). Occupational violence and aggression experienced by nursing and caring profession. *Journal of Nursing Scholarship*.

[60] Small C. R., Porterfield S., Gordon G. (2015). Disruptive behavior within the workplace. *Applied Nursing Research*.

[61] Tong M., Schwendimann R., Zúñiga F. (2017). Mobbing among care workers in nursing homes: a cross-sectional secondary analysis of the Swiss Nursing Homes Human Resources Project. *International Journal of Nursing Studies*.

[62] Welch C. E., Hodgson M. J., Haberfelde M. (2013). Impact of medical center complexity on veterans health administration nursing staff incidence rates for reported assaults. *Work*.

[63] Ruiz-Hernández J. A., Sánchez-Muñoz M., Jiménez-Barbero J. A. (2019). User violence in mental health services: adaptation of an instrument. Healthcare-Workers’ aggressive behavior scale-users-mental health version (HABS-U-mh). *PLoS One*.

[64] Liu J., Gan Y., Jiang H. (2019). Prevalence of workplace violence against healthcare workers: a systematic review and meta-analysis. *Occupational and Environmental Medicine*.

[65] Johnson S. L. (2009). International perspectives on workplace bullying among nurses: a review. *International Nursing Review*.

[66] Leymann H. (1996). The content and development of mobbing at work. *European Journal of Work & Organizational Psychology*.

[67] Ozturk H., Sokmen S., Yılmaz F., Cilingir D. (2008). Measuring mobbing experiences of academic nurses: development of a mobbing scale. *Journal of the American Academy of Nurse Practitioners*.

[68] Nelson S., Leslie K., Mccormick A. (2023). Workplace violence against nurses in Canada: a legal analysis. *Policy, Politics, & Nursing Practice*.

[69] Vessey J. A., DeMarco R., DiFazio R. (2010). Bullying, harassment, and horizontal violence in the nursing workforce: the state of the science. *Annual Review of Nursing Research*.

[70] Brooks I., Morphet J. (2021). The defining characteristics of newly graduated nurses – a Delphi study. *Nurse Education in Practice*.

[71] Zhang X., Xiong L. (2019). Impact of nurse horizontal violence and coping strategies: a review. *Yangtze Medicine*.

[72] Ajoudani F., Baghaei R., Lotfi M. (2019). Moral distress and burnout in Iranian nurses: the mediating effect of workplace bullying. *Nursing Ethics*.

[73] Rush K. L., Adamack M., Gordon J., Janke R. (2014). New graduate nurse transition programs: relationships with bullying and access to support. *Contemporary Nurse*.

[74] Shi Y., Guo H., Zhang S. (2018). Impact of workplace incivility against new nurses on job burn-out: a cross-sectional study in China. *BMJ Open*.

[75] Chang H. E., Cho S.-H. (2016). Workplace violence and job outcomes of newly licensed nurses. *Asian Nursing Research*.

[76] Magnavita N., Heponiemi T. (2011). Workplace violence against nursing students and nurses: an Italian experience. *Journal of Nursing Scholarship*.

[77] Jafree S. R. (2017). Workplace violence against women nurses working in two public sector hospitals of Lahore, Pakistan. *Nursing Outlook*.

[78] Hinchberger P. A. (2009). Violence against female student nurses in the workplace. *Nursing Forum*.

[79] Hopkins M., Fetherston C. M., Morrison P. (2014). Prevalence and characteristics of aggression and violence experienced by Western Australian nursing students during clinical practice. *Contemporary Nurse*.

[80] Tian L., Zhang Y., Li X. (2019). Research on the resilience of Chinese nursing students to workplace vertical violence in clinical practice. *Nurse Education in Practice*.

[81] Escribano R. B., Beneit J., Luis Garcia J. (2019). Violence in the workplace: some critical issues looking at the health sector. *Heliyon*.

[82] Kafle S., Paudel S., Thapaliya A., Acharya R. (2022). Workplace violence against nurses: a narrative review. *Journal of Clinical and Translational Research*.

[83] Spector P. E., Zhou Z. E., Che X. X. (2014). Nurse exposure to physical and non-physical violence, bullying and sexual harassment: a quantitative review. *International Journal of Nursing Studies*.

[84] Schablon A., Wendeler D., Kozak A., Nienhaus A., Steinke S. (2018). Prevalence and consequences of aggression and violence towards nursing and care staff in Germany- A survey. *International Journal of Environmental Research and Public Health*.

[85] World Health Organization (WHO) (2018). Violence and injury prevention. http://www.who.int/violence_injury_prevention/violence/worplace/en/.

[86] Varghese A., Joseph J., Vijay V. R. (2022). Prevalence and determinants of workplace violence among nurses in the South‐East Asian and Western Pacific Regions: a systematic review and meta‐analysis. *Journal of Clinical Nursing*.

[87] Bernardes M. L. G., Karino M. E., Martins J. T., Okubo C. V. C., Galdino M. J. Q., Moreira A. A. O. (2020). Workplace violence among nursing professionals. *Revista Brasileira de Medicina Do Trabalho*.

[88] Liu X., Yang H., Hu Y. (2022). Incidence of workplace violence against nurses among Chinese hospitals: a meta‐analysis. *Journal of Nursing Management*.

[89] Dahlke S., Baumbusch J. (2015). Nursing teams caring for hospitalised older adults. *Journal of Clinical Nursing*.

[90] Jacob E. R., McKenna L., D’Amore A. (2016). Educators’ expectations of roles, employability and career pathways of registered and enrolled nurses in Australia. *Nurse Education in Practice*.

[91] McKenna L., Wood P., Williams A. (2019). Scope of practice and workforce issues confronting Australian Enrolled Nurses: a qualitative analysis. *Collegian*.

[92] MacLeod M., Kosteniuk J., Penz K. (2019). Rural and remote licensed practical nurses’ perception of working below their legislated scope of practice. *Canadian Journal of Nursing Leadership*.

[93] Mueller C., Duan Y., Vogelsmeier A., Anderson R., McConnell E., Corazzini K. (2018). Interchangeability of licensed nurses in nursing homes: perspectives of directors of nursing. *Nursing Outlook*.

[94] Ramacciati N., Gili A., Mezzetti A., Ceccagnoli A., Addey B., Rasero L. (2019). Violence towards emergency nurses: the 2016 Italian national survey—a cross‐sectional study. *Journal of Nursing Management*.

[95] Lu L., Lok K.-I., Zhang L. (2019). Prevalence of verbal and physical workplace violence against nurses in psychiatric hospitals in China. *Archives of Psychiatric Nursing*.

[96] O’Keefe D. (2016). *Staff Experience High Rates of Aggression in Aged Care*.

[97] Johnson J., Cameron L., Mitchinson L. (2019). An investigation into the relationships between bullying, discrimination, burnout and patient safety in nurses and midwives: is burnout a mediator?. *Journal of Research in Nursing*.

[98] Song J., Mcdonald C. (2021). Experiences of New Zealand registered nurses of Chinese ethnicity during the COVID-19 pandemic. *Journal of Clinical Nursing*.

[99] National Commission to Address Racism in Nursing (2022). *Racism’s Impact In Nursing*.

[100] Thurman W. A., Johnson K. E., Sumpter D. F. (2019). Words matter: an integrative review of institutionalised racism in nursing literature. *Advances in Nursing Science*.

[101] Kaiser J. A. (2017). The relationship between leadership style and nurse-to-nurse incivility: turning the lens inward. *Journal of Nursing Management*.

[102] Shore L. M., Cleveland J. N., Sanchez D. (2018). Inclusive workplaces: a review and model. *Human Resource Management Review*.

[103] American Organization of Nurse Executives (2015). AONE and ENA develop guiding principles on mitigating violence in the workplace. *Journal of Emergency Nursing*.

[104] Ceravolo D. J., Schwartz D. G., Foltz-Ramos K. M., Castner J. (2012). Strengthening communication to overcome lateral violence. *Journal of Nursing Management*.

[105] Kiprillis N., Gray R., Robinson E., McKenna L. (2022). Prevalence of horizontal violence of nurses in their first year of practice: a systematic review. *Collegian*.

